# Highly Sensitive and Multifunctional Tactile Sensor Using Free-standing ZnO/PVDF Thin Film with Graphene Electrodes for Pressure and Temperature Monitoring

**DOI:** 10.1038/srep07887

**Published:** 2015-01-20

**Authors:** James S. Lee, Keun-Young Shin, Oug Jae Cheong, Jae Hyun Kim, Jyongsik Jang

**Affiliations:** 1 World Class University program of Chemical Convergence for Energy & Environment, School of Chemical and Biological Engineering, Seoul National University, 151-742, Korea; 2 Manufacturing Technology Team, Infra Technology Service Center, Device Business, Samsung Electronics, San #16 Banwol-Dong, Hwasung-City, Gyeonggi-Do, Korea

## Abstract

We demonstrate an 80-μm-thick film (which is around 15% of the thickness of the human epidermis), which is a highly sensitive hybrid functional gauge sensor, and was fabricated from poly(vinylidene fluoride) (PVDF) and ZnO nanostructures with graphene electrodes. Using this film, we were able to simultaneously measure pressure and temperature in real time. The pressure was monitored from the change in the electrical resistance via the piezoresistance of the material, and the temperature was inferred based on the recovery time of the signal. Our thin film system enabled us to detect changes in pressure as small as 10 Pa which is pressure detection limit was 10^3^-fold lower than the minimum level required for artificial skin, and to detect temperatures in the range 20–120°C.

Electronic skin (e-skin) mimics some of the functionality of human skin, aiming to provide sensory responses to mechanical, thermal, chemical, biological and optical stimuli. Therefore, multifunctional electronic sensors are of great interest for medical and industrial applications. In particular, thin-film systems have been investigated for use in sensor devices, due to the convenient, low-cost processing techniques and flexibility. Moreover, the development of a flexible thin-film device that can replace the epidermis has important applications in the treatment of injuries. Much effort has been devoted to enhancing the performance of pressure-sensing films. Integrated, strain-gauge sensors have been reported by Kahp-Yang Shu *et al.*,^1^ that could detect pressure, shear and torsion. In contrast, relatively little attention has been paid to sensing of combined external stimuli in a single device, such as sensing mechanical strain and temperature. It is not straightforward to design a system that can discriminate among different stimuli, because the fabrication of such multifunctional sensors typically involves integration of a number of organic and inorganic devices using hybrid matrix arrays and circuit elements, which involves complex, expensive processes.^2^,^3^,^4^,^5^,^6^,^7^,^8^,^9^,^10^,^11^,^12^,^13^ Thus, it is still challenging to realize a sensor that enable to detect multiple stimuli accurately and simultaneously.

To date, much effort has been devoted to investigating the piezoelectric and pyroelectric properties of thin films for fabrication of multifunctional sensors. The piezoelectric and pyroelectric properties of thin films enable fabrication of multifunctional sensors. Reverse-piezoelectricity can respond to an applied mechanical displacement by inducing an electric potential and pyroelectricity.^14^,^15^,^16^,^17^,^18^,^19^ An example of such a material is poly(vinylidene fluoride) (PVDF), which has attracted much interest as a next-generation piezoelectric and pyroelectric material because of its light weight, flexibility, low power consumption, and non-toxicity.^20^,^21^,^22^,^23^,^24^ We have previously demonstrated the piezoelectric effect of PVDF for application as an acoustic acuator.^4^ The enhanced permittivity, which is related to the polarization and dipole moment of PVDF, is key factor for improving the piezoelectric and pyroelectric properties of PVDF. For this reason, the semiconductor zinc oxide (ZnO) is of interest, as it may increase the piezoelectric response, is thermally stable, and may increase the permittivity of PVDF^25^,^26^. Therefore, a hybrid PVDF matrix with a ZnO nanofiller may enable fabrication of a hybrid piezoelectric/pyroelectric sensor.

Furthermore, piezoresistive and pyroresistive sensing, which transduces mechanical displacement and temperature signals into an electrical signal, may be a valuable and simple monitoring method for small structures^1^. In particular, a change in the electron mobility or resistance can be measured using a transistor structure.

Here we report a highly sensitive hybrid functional gauge sensor using a PVDF thin film and vertically grown ZnO nanorods. As electrodes, a graphene was utilized which is derived from the vacuum-assisted reduction of graphene oxide (GO) film by inkjet printing. To our knowledge, this is the first experimental demonstration of the detection of two different independent stimuli (i.e., pressure and temperature) simultaneously and in real time. The pressure was inferred from the change in the electrical resistance via the piezoresistance of the material, and the temperature was determined based on the recovery time of the signal. The morphology of the ZnO nanostructures was controlled to maximize the response of the device, and the output signals were monitored while controlling the pressure and temperature to calibrate the device.

## Results

### Fabrication of a PVDF- and ZnO-based sensor

**Figure 1:**
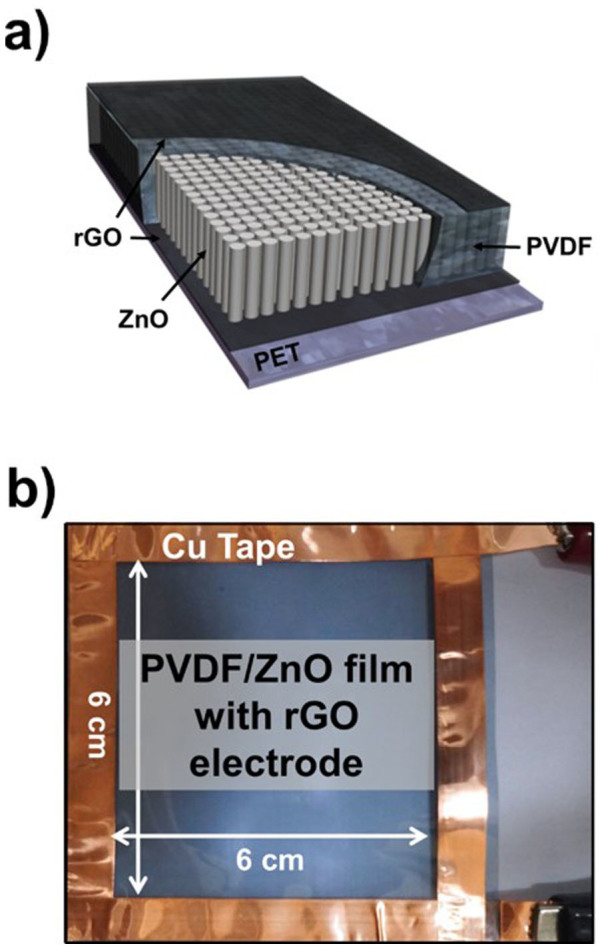
(a) Schematic diagram and (b) a photograph of the device consisting of the ZnO/PVDF composite film and rGO electrodes.

Fig. 1a and b show a schematic diagram and photograph of the flexible multilayer device. A large areal density of ZnO nanorods was embedded in the PVDF on a reduced graphene oxide (rGO)-treated flexible polyethylene terephthalate (PET) thin film. First, exfoliated GO aqueous ink was modified on the flexible PET substrate via inkjet printing, and reduced for use as an electrode^27^. The ZnO nanorods were then grown, followed by a hydrothermal treatment, as shown in Fig. 1a. The 20-wt% PVDF solution was dissolved in a 1:1 mixture of acetone and dimethylformamide (DMF), and was spin-coated onto the ZnO nanorods, which then coagulated^28^. A poling process was then carried out under a strong constant electric field of 300 kV/cm at 90°C to induce piezoelectricity and pyroelectricity in the PVDF^4^. A top electrode of conductive ink was coated onto the PVDF/ZnO nanorod composite film using inkjet printing.

### Characterization of the rGO electrode

**Figure 2:**
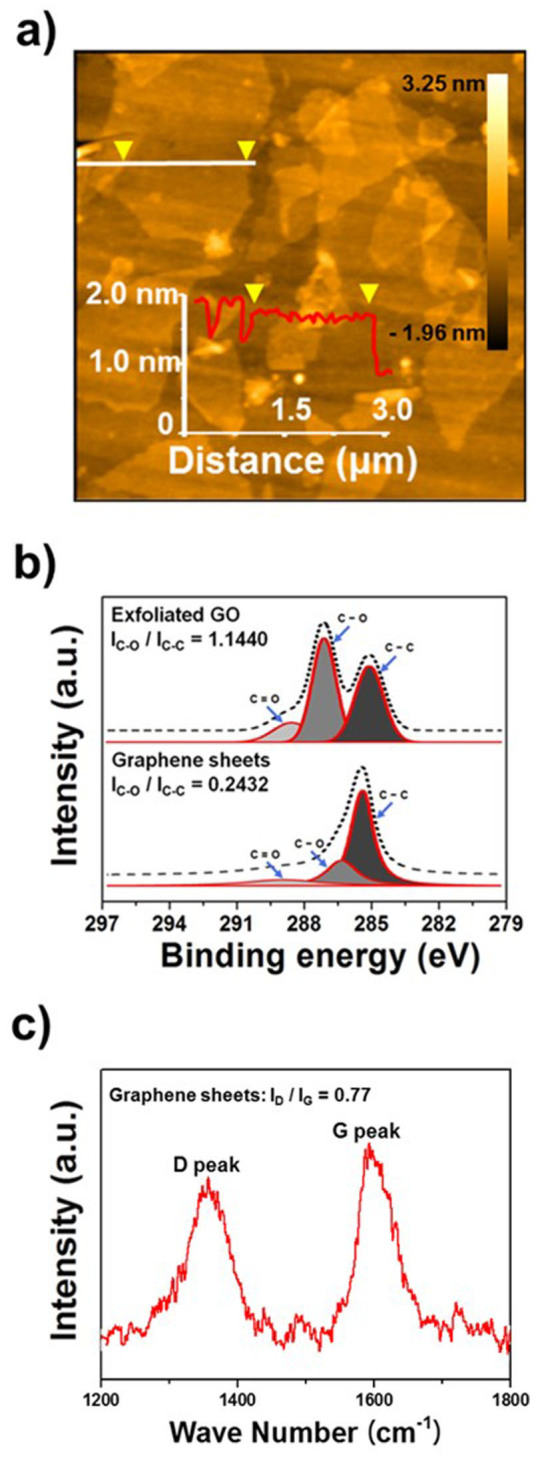
(a) AFM image of the exfoliated GO solution. (b) XPS spectrum with fitted lines of the pristine and rGO solution for C–C, C–O and C = O bonds. (c) Raman spectra of the rGO electrodes.

To fabricate the conductive ink electrode, an aqueous solution GO was synthesized using the Hummers and Offeman method^29^,^30^,^31^. Fig. 2a shows an atomic force microscopy (AFM) image of the fabricated GO ink, which was a few microns thick, and was composed of 2–3-nm-thick bilayers, as well as some multilayers (>3 nm). To demonstrate the reduction of GO solution to graphene, X-ray photoelectron spectroscopy (XPS) and Raman spectroscopy analysis were carried out. Fig. 2b shows deconvoluted C 1s XPS spectra of the inkjet-printed GO and the reduced GO (rGO) sheets^18^,^19^. The C 1s signal of GO has three main components: the C = C and C−C bond vibrations in the in aromatic rings (285.0 eV), C−O (286.5 eV) in epoxy or hydroxyl groups, and C = O (288.5 eV) peaks originating from carbonyl and carboxy groups. The ratio *I_C−O_*/*I_C−C_* decreased from 1.1440 (GO) to 0.2432 (rGO). In addition, a Raman spectrum of the printed rGO sheets on the substrate film is shown in Fig. 2c. The Raman spectra of rGO clearly exhibited the characteristic peaks of the D band (~1350 cm^−1^), which indicate the typical defects ascribed to structural edge effects, such as epoxides covalently bonded to the base plane, the G band (~1580 cm^−1^), which indicates a graphite carbon structure, and the ration of *I_D_/I_G_* was 0.77^30^,^31^.

### Characterization of the ZnO nanostructures

**Figure 3:**
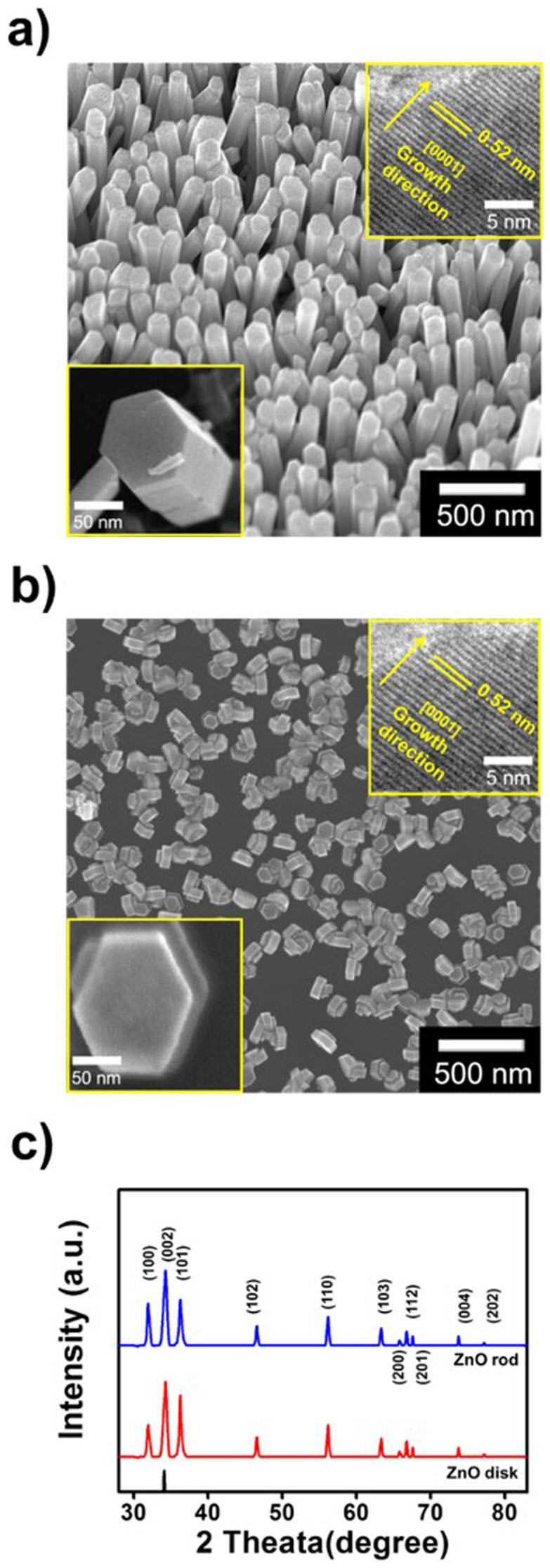
SEM images of (a) the ZnO nanorods and (b) the ZnO nanodisks. (c) XRD spectra to investigate the crystal structure of the ZnO nanorods and disks.

The ZnO nanostructures were fabricated using a seed solution zinc acetate (Zn(CH_3_COO))_2_ dissolved in ethanol, which was spin-coated onto the rGO-treated PET film. The ZnO nanorods were grown from a mixture of zinc nitrate hexahydrate (Zn(NO_3_)_2_·6H_2_O), hexamethylenetetramine and water at 90°C. The morphology of the ZnO nanorods was controlled by varying the time and concentration of the solution of growth precursor. During the early stages of the hydrothermal method, ZnO nucleates spontaneously from the ionized Zn(OH)_4_^2−^ seed, to form a hexagonal nanostructure in the mixture, which then initiates the growth of one-dimensional (1D) nanorods by increasing the temperature and concentration. As shown in Figs. 3a and b, field-emission scanning electron microscopy (FE-SEM) images reveal that a high density of ZnO nanorods (1.42 × 10^10^ cm^−2^) and disks were formed. Furthermore, high-resolution transmission electron microscopy (HR-TEM) images show that the ZnO nanorods and nanodisks had a well resolved single-crystalline wurtzite structure, with a lattice constant of 0.52 nm in the [0001] growth direction^32^,^33^. This indicates that they formed a uniform hexagonal crystal structural without crystalline defects (see inset Fig. 3a and b). The length of the ZnO nanorods was ~300 nm, and the diameter was ~85 nm; the ZnO nanodisks were ~30 nm in length and ~100 nm in diameter. To investigate the crystal structure of the ZnO nanorods and nanodisks, X-ray diffraction (XRD) analysis was carried out, as shown in Fig. 3c. All of the diffraction peaks could be clearly indexed to the hexagonal ZnO with a lattice constant of *c* = 0.5206 nm, which is in good agreement with that of ZnO along the *c*-axis^32^.

### Dielectric properties of the PVDF/ZnO device

**Figure 4:**
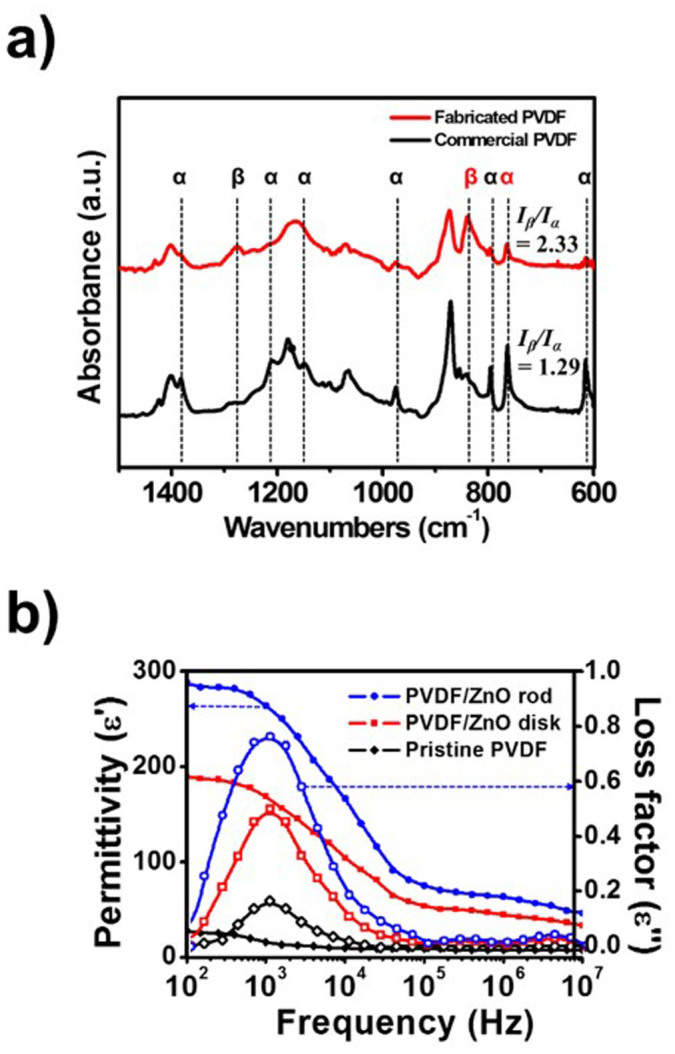
(a) FTIR spectra of PVDF film fabricated as part of this work, and a commercially available PVDF film. The crystalline phases are indicated. (b) The permittivity and losses of the tactile sensor fabricated with PVDF and different ZnO nanostructures, as well as the PVDF thin film and the commercially available PVDF thin film.

The piezoelectric and pyroelectric properties of PVDF are strongly dependent on the *β*-phase content, owing to large net dipole moment, which originates from the all-trans structure^4^,^34^. Therefore, Fourier transform infrared (FTIR) spectroscopy was carried out to investigate the strong dielectric potential, based on the ratio of the *α* and *β* phases (*I_β_/I_α_*), and the fraction of the *β* phase, as shown in Fig. 4a. Assuming that the IR absorption follows the Beer-Lambert law, *β* content in the PVDF can be measured from: 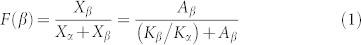
where *A_α_* and *A_β_* are the absorbance at 766 and 840 cm^−1^, respectively, and *K_α_* = 6.1 × 10^4^ cm^2^/mol and *K_β_* = 7.7 × 10^4^ cm^2^/mol are the absorption coefficients of *α* and *β* phases at the respective wavenumbers^4^. The PVDF thin film exhibited *I_β_/I_α_* = 2.33, which corresponds to an 85% *β* phase. (For comparison, commercially available PVDF exhibited *I_β_/I_α_* = 1.29, which corresponds to a 73% *β* phase content.)

Generally, PVDF with a high *β*-phase content exhibits enhanced piezoelectric and pyroelectric responses, due to fact that the dipoles are aligned. Also, Fig. S1. shows that observed polarized optical microscopy morphology (POM) of the crystal growth of PVDF with various ZnO fillers. All samples were isothermally crystallized at 170°C and maintained for 240 s, and it cooled down to observe crystal growth morphology. During poling process of film, high external voltage was applied on the film and polarized ZnO induced β phase of PVDF. Thus, regardless of ZnO morphology, it is clarified that ZnO acts as nuclei for PVDF crystallization. Therefore, we may expect favorable permittivity and losses of sensors fabricated using various ZnO and PVDF thin films. The dielectric properties of PVDF with the embedded ZnO nanorods can be estimated using the Havriliak-Negami and Fourier transfer relationship; i.e.:
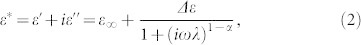
Where ε′ is the dielectric constant, ε″ is the dielectric loss; the permittivity is therefore given by: 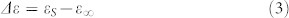
where *ε_s_* is the static permittivity (i.e., when lim*_ω_*_→0_ε*(*ω*)), and *ε*_∞_ is the high-frequency permittivity (i.e., when lim*_ω_*_→∞_ε*(*ω*))^4^,^35^,^36^.

The measured permittivities of the samples are listed in Fig. 4b. and Table S1. In general the permittivity of a dielectric is larger at lower frequencies than at higher frequencies, and embedding the ZnO in the PVDF significantly increased the dielectric constant. In particular, the ZnO nanorods possessed a greater dielectric constant than the ZnO disks, which is attributable to the larger aspect ratio of the ZnO nanorods, which resulted in a larger energy barrier.

The relaxation time *λ* is related to the gradient of the loss factor that is associated with the interfacial polarization response of the dielectric material. The relaxation time is given by:
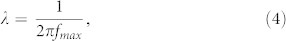
where *f_max_* is the frequency of the peaks in the loss spectrum^36^. We find a relaxation time of *λ* = 159 for the PVDF, *λ* = 130 μs for the ZnO nanodisks, and *λ* = 121 μs for the ZnO nanorods (see Table S1). Based on these dielectric parameters, the inclusion of ZnO in the PVDF film significantly increased the permittivity and reduced the polarization response time of the PVDF-based sensor devices. Since ZnO nanorods with PVDF possessed a higher dielectric constant, the concentration of seed solution was controlled to investigate effects of ZnO rods density. Approximately 30 wt% of zinc acetate dissolved seed solution grow 1.42 × 10^10^ cm^−2^, and 25 and 35 wt% represent 0.68 × 10^10^ cm^−2^ and 2.54 × 10^10^ cm^−2^ of ZnO rods. As a result, 25 wt% of zinc acetate solution sparsely grow ZnO rods compare to 30 wt% of seed solution which result in reduced dielectric properties. Additionally, 35 wt% of seed solution limits dielectric properties of fabricated sample because exceeding density of ZnO rods caused aggregation between rods (Table S2).

### Detecting pressure and temperature

**Figure 5:**
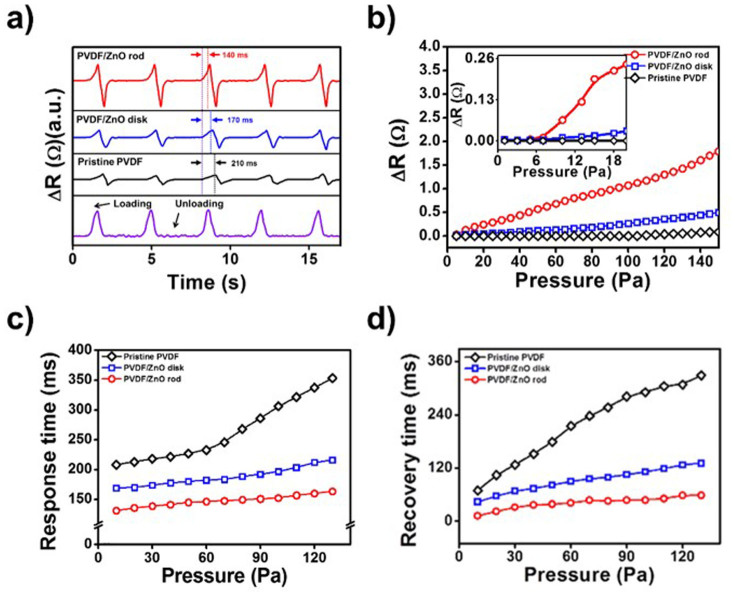
The change in resistance of the pristine PVDF, and the PVDF/ZnO composite films. (a) In response to an applied pressure of 30 Pa, showing the response time. (b) The sensitivity of the films at 20°C, using various Pt weights. (c) The response and (d) recovery times of the PVDF film, the ZnO nanodisk/PVDF film, and the ZnO nanorod/PVDF with various pressures.

To compare the pressure-sensing properties of the PVDF and ZnO/PVDF composite films, pressure stimuli were used, and the performance was measured from the change in the electrical resistance; i.e., Δ*R* = *R_loading_*−*R_Unloading_* (Fig. S2). The assembled device had an overlap area of 6 × 6 cm^2^, and was measured with a gradual increase in pressure. Fig. 5a shows how the composite device formed of PVDF, ZnO nanorods and ZnO nanodisks responded to application of a constant pressure. The surface of compressed ZnO nanorods generates a negative potential, and the electrical contact with the rGO electrode layer and the forward bias of the Schottky barrier at the interface results in a change in the resistance of the device (Fig. S3). A difference in the response Δ*R* to the same pressure was observed between devices, and the PVDF/ZnO nanorod composite device exhibited the greatest signal in response to a given stimulus. Because of the 1D structure of the vertically grown ZnO nanorods, they generate an enhanced piezoelectric response to mechanical displacement than the ZnO nanodisks. The larger aspect ratio and permittivity lead to a change in the electrical resistance in response to even a slight deflection of the ZnO nanorods.

The pressure-sensing capability of the system was evaluated from the response Δ*R*, as shown in Fig. 5b. The smallest detectable pressure using the ZnO nanorod/PVDF film was 10 Pa (giving Δ*R* = 0.062 Ω), which corresponds to 1 mg mm^−2^, which is 1000-fold more sensitive than the minimum requirements for artificial skin^3^,^37^,^38^,^39^,^40^. The response times of the output signals from ZnO nanorod/PVDF device were obtained under various pressures at 20°C. The recovery times exhibited similar as shown in Figs. 5c and d. Additionally, three different conducting material of rGO, CNT and PEDOT: PSS were candidate to demonstrate pressure sensitivity as a function of various electrode (Fig. S4.). The sheet resistances of commercial poly(3,4-ethylenedioxythiophene):poly(styrenesulfonate) (PEDOT:PSS) (Sigma-Aldrich) and carbon nanotubes (CNT) (Sigma-Aldrich), and synthesized rGO were 34,10 and 0.7 kΩ/sq. As a result, PVDF/ZnO rods with rGO electrode has highest sensitivity under constant pressure compare to other electrode utilized device.

**Figure 6:**
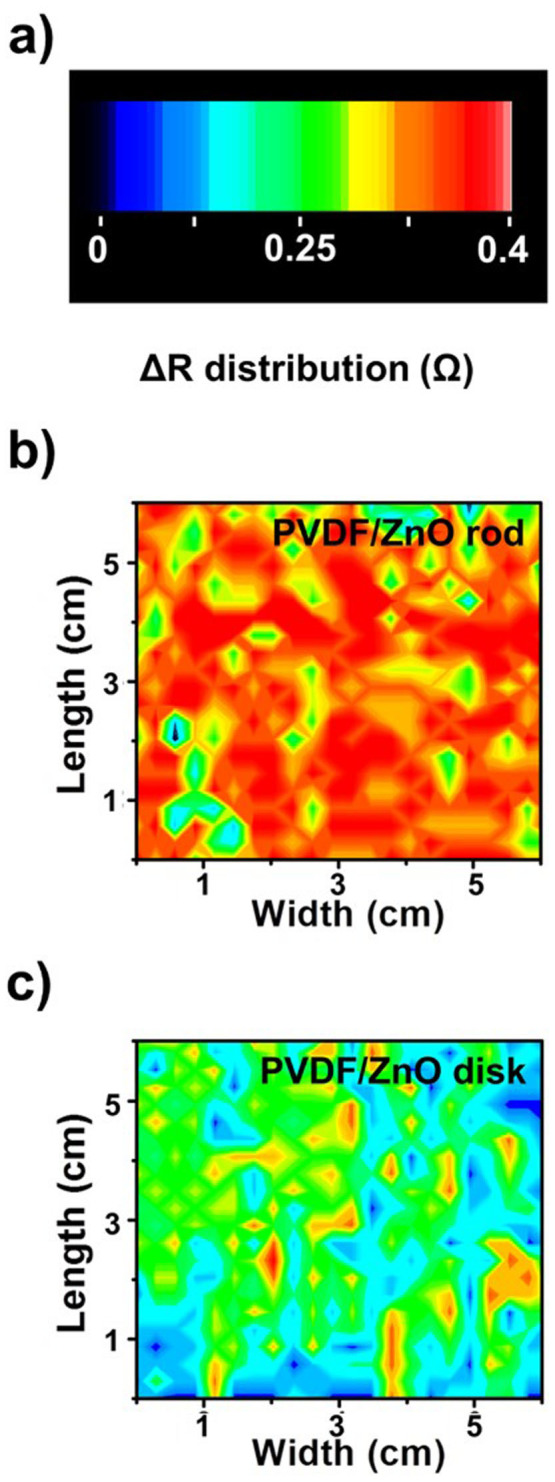
(a) The spatial variation in the change in resistance. The effective area of fabricated device are 6 × 6 cm^2^. (b) The ZnO nanorod/PVDF film exhibited a higher pressure sensitivity than (c) the ZnO nanodisk/PVDF film. The film was divided into 144 regions (0.5 × 0.5 cm^2^) and Pt weights were used to apply a pressure of 30 Pa at each division, one at a time.

To investigate the spatial resolution of the pressure-sensing ability, ZnO/PVDF devices were fabricated, as shown in Fig. 6. The film was divided into 144 regions, each 0.5 × 0.5 cm^2^, and Pt weights were used to apply a pressure of 30 Pa at each division, one at a time. The output signal was measured from the change in the resistance using a pseudocolor plot, as shown in Fig. 6a. The two-dimensional (2D) intensity profiles of the ZnO nanorod/PVDF film are shown in Fig. 6b, and the 2D intensity profile of that formed using the ZnO nanodisk/PVDF film are shown in Fig. 6c. The distribution of the operational device area containing the ZnO nanorods in the PVDF thin film was 96%, and that containing the ZnO nanodisks was 77%. As the areal density of ZnO nanorods was larger, and significant aggregation did not occur, in contrast to the ZnO nanodisks, and so we may expect a more uniform pressure sensitivity with the ZnO nanorod/PVDF composite film.

**Figure 7:**
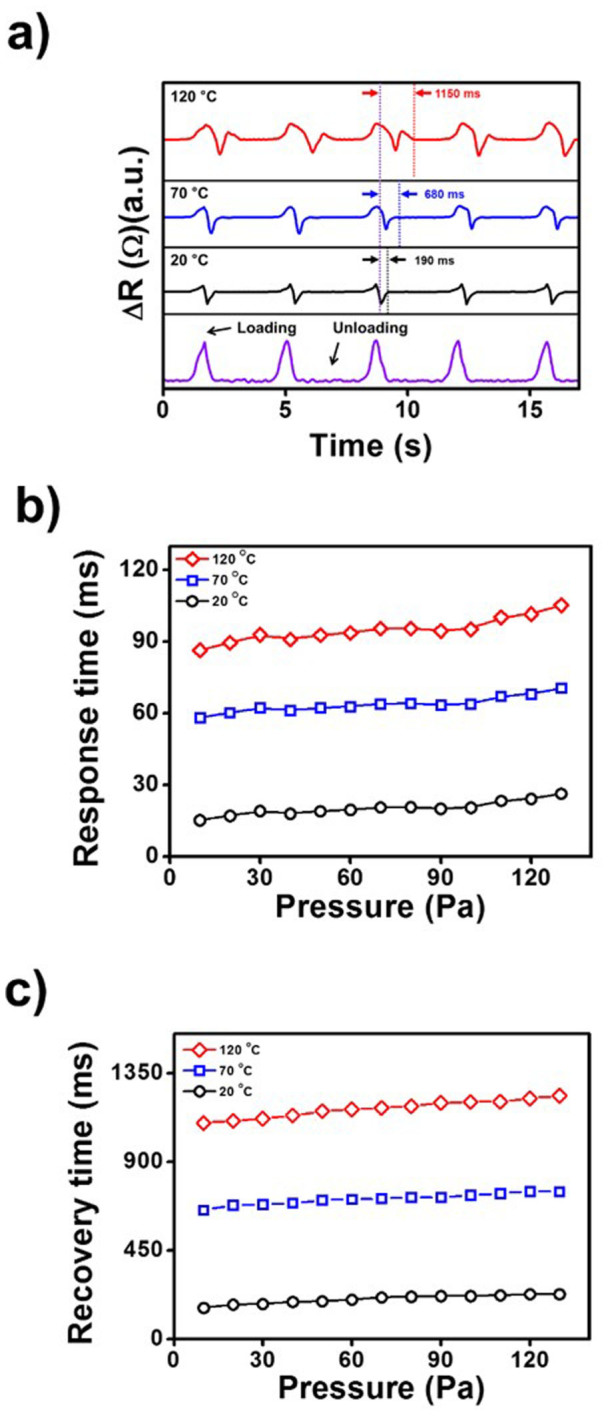
(a) The output resistance of the PVDF/ZnO nanorod device at various temperatures, 20, 70, and 120°C, under constant pressure of 30 Pa in the center of device. (b) The response to different pressures and (c) the recovery time at various temperatures.

The device composed of the ZnO nanorod/PVDF composite was used to measure the output signals with various temperatures and applied pressures. To evaluate the pyroelectric response, the Pt weight, which applied a pressure of 30 Pa, were applied at 20, 70, and 120°C in the center of device, as shown in Fig. 7a. We found that the response was slower, and the recovery time longer, at higher temperatures. This is because the pyroelectricity originated from the PVDF, and the thermal energy released from the heated weight on the surface of PVDF thin film resulted in slower motion of the dipoles^15^,^34^,^41^,^42^,^43^,^44^. Loading at 20°C resulted in a recovery time of 190 ms, loading at 70°C resulted in a recovery time of 680 ms, and loading at 120°C in a recovery time of 1150 ms.

As shown Figs. 7b and 7c, reproducible responses to changes in temperature and pressure were obtained. The independence of Δ*R* and the recovery time enabled measurement of pressure and temperature independently. Compare to obtained respons and recovery time, it is found that recovery time has wide delayed time range, 1150 ms at 120°C with 30 Pa whereas delayed response time observes 92 ms. Additionally, the analyzed uniformity of temperature-sensing at the fabricated ZnO nanorods embedded PVDF thin film was 94% (Fig. S5). Considering these results, it is worth nothing that investigation of resistance difference make it feasible to measure the weight of pressure, and delayed recovery time play a pivotal role in determining the temperature of target object.

### Application as tactile sensor

**Figure 8:**
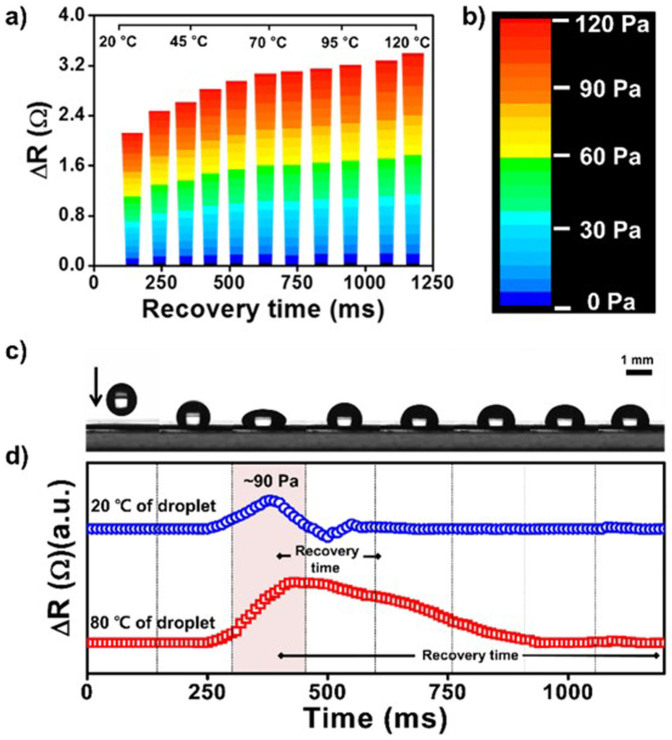
The relationship between external pressure and the temperature of the object placed on the device to induce a pressure response. (a) The change in the resistance and recovery time. (b) Pseudocolor plots showing the applied pressure on the PVDF/ZnO nanorod film. (c) The response to impact of a droplet with an unknown temperature. (d) The recovery time shows that the temperatures of the droplets were 20°C and 80°C, and the pressure was 90 Pa.

Mapping data of the two independent factors was evaluated based on external pressure loaded simultaneously and the temperature of the device. As shown in Figs. 8a and 8b, the applied pressure changed the resistance. Increasing the temperature of the object placed on the then film to apply the pressure increased the recovery time of the signal, and so the temperature of object could be calculated based on the recovery time. To investigate the scope for simultaneous pressure and temperature sensing in real time, a 15-μL mineral oil droplet dropped from an initial height of 1.5 cm was measured under a low-noise environment, as shown in Fig. 8c. Data were collected during the initial impact, and during the time when the droplet rebounded from the surface, and impacted again, bouncing on the surface over a period of 1.2 s, as shown in Fig. 8d. The maximum signal at 20°C following the impact of the droplet onto the film was Δ*R* = 1.62 Ω, which is equivalent to a pressure of 90 Pa; the recovery time was 180 ms. The signal when a droplet with an unknown temperature was dropped from the same height was monitored. The maximum response was Δ*R* = 1.61 Ω. However, recovery time was 815 ms, which corresponds to a droplet temperature of 80°C. The difference in the two peak shapes is attributable to the difference in the sample temperature of each droplet. The 20°C droplet that impacted on the film did not heat the film, because they were at an identical temperature. Therefore, the bouncing movement of a droplet can be detected after its first impact at 510 ms. However, the 80°C droplet was able to transfer both kinetic energy and heat to the film. For this reason, the recovery time was prolonged. Therefore, this system enables independent measurement of pressure and temperature based on the magnitude of the change in the resistance and the recovery time of the signal.

## Discussion

We have demonstrated a highly sensitive and multifunctional sensor using a PVDF/ZnO nanorod composite thin film. Existing approaches focus on improving the sensitivity, whereas here we could measure the pressure and temperature independently. The strategy used in the design and fabrication of ZnO nanorods embedded in the PVDF thin film represents a significant advance in the state of the art of nanotechnology fabrication methods. The high sensitivity to pressure and the ability to independently sense temperature are attributable to the properties of the vertically grown ZnO nanorods and enhanced *β*-phase component of the PVDF film. The morphology of ZnO nanostructures was either disks or rods (Fig. 3), and could be controlled by varying the process conditions. As a result, the dielectric behavior of PVDF was improved owing to the piezoelectric barrier provided by the ZnO nanorods (Fig. 4).

The composite sensing film exhibited high sensitivity and accuracy, and a real-time response. Using the ZnO nanorods embedded in the PVDF thin film, the pressure sensor could respond to very small pressures, with a minimum detectable pressure of 10 Pa. Moreover, we were able to detect temperatures in the range 20–120°C. Pyroelectricity induced by the surface of the PVDF film resulted in variation in the output recovery time, from which we could infer the temperature of target object (see Fig. 8).

In conclusion, we fabricated composite ZnO/PVDF thin films, and used these structures to independently measure the pressure applied to the film and the temperature of the object used to apply the pressure. The pressure detection limit was 10^3^-fold lower than the minimum level required for artificial skin, could be monitored in real time, and the temperature could be inferred from the recovery time of the signal. This favorable performance of the e-skin represents a significant advance over the current state of the art, and has potential applications in biorobotic fields.

## Methods

### Materials

Graphite was purchased from Sigma-Aldrich. The graphene electrodes were printed using a commercially available office inkjet printer (Canon Pixima Ip1300). The polyethylene terephthalate (PET) film, polyvinylidene fluoride (PVDF) pellets (with a molecular weight of 275,000, determined via gel permeation chromatography), zinc acetate dihydrate, zinc nitrate hexahydrate, hexamethylenetertramine and ethanol were obtained from Sigma−Aldrich. PVDF film was obtained from the Fils Corporation (South Korea).

### Synthesis of GO dispersion for inkjet printing

GO was synthesized from natural graphite via the modified Hummers and Offeman method, as described by Kovtyukhova and colleagues. Synthesized purified GO suspensions were exfoliated in water by sonication for 1.5 h to avoid blocking the nozzle. The obtained brown dispersion was then washed for 4 cycles of centrifugation at 5,000 rpm to remove any unexfoliated GO.

### Modification of the inkjet printer

The ink cartridge (printer head) was disassembled and washed several times using ethanol and distilled water after removing all the inks. The exfoliated GO was dispersed distilled water at 0.1 wt%, and was injected into the modified cartridge. The sealed ink cartridge was then placed in the printer body for use in further experiments.

### Preparation of ZnO

Zinc acetate was dissolved in 100 mL of ethanol, spin-coated onto the rGO-treated PET film, placed in an oven at 100°C, and dried for 30 min to create a seed layer for subsequent growth of the ZnO nanostructures. A 500-mL solution of 0.05 M hexamethylenetetramin and 0.05 M zinc nitrate hexahydrate and de-ionized water was then coated onto the substrate, and the ZnO nanostructures were grown at 90°C for 3–7 h to control the morphology of ZnO nanostructures.

### Preparation of the PVDF thin film

PVDF was dissolved in a 1:1 mixture of DMF and acetone. The solution was stirred at 60°C for 12 h and dropped onto the substrate containing the ZnO nanostructures. Following spin-coating at 1200 rpm for 150 s, the film was poled under an electric field of 300 kV/cm at 90°C.

### Characterization

HR-TEM images were captured using a JEOL JEM-3010. Field-emission scanning electron microscopy (FE-SEM) (JEOL JSM-6700F, Japan) and a FTIR spectroscopy (Bomem MB 100, USA) were also used. Atomic force microscopy (AFM) (Nanoscope Шa, Digital instruments, USA) was used to image the surface topography. AFM measurements were carried out in tapping mode with silicon tips at a resonance frequency of 320 kHz. The electrical resistance was measured using a Keithley 2400 source meter at 25°C using a four-probe method. Raman spectra were recorded using a LabRAM HR (Horiba, Japan) with 1064-nm laser excitation. For the analysis of detailed force responses, a computer-based user interface and a micro pressure sensor (FT-S270, Nano Science Instrument) with nanoscale-controlled stage by piezoelectric stepping positioner (SLC-1730, Nano Science Instrument) were used to apply an external pressure. Moreover, direction of stepper to apply pressure was vertically loaded and unloaded in order to avoid distortion output signals by torsion or sheer stress.

## Author Contributions

J.S.L and K.Y.S. contributed equally to this work; J.S.L., K.Y.S., O.J.C. and J.H.K. designed and performed the experiments, and authored the manuscript; J.S.L. and K.Y.S. contributed to data collection and theoretical analysis; J.J. planned and supervised the project; All authors edited the manuscript.

## Supplementary Material

Supplementary Information supplementary information
