# Supplementary material for: Highly Sensitive and Multifunctional Tactile Sensor Using Free-standing ZnO/PVDF Thin Film with Graphene Electrodes for Pressure and Temperature Monitoring
Source: Sci Rep. 2015 Jan 20;5:7887. doi: 10.1038/srep07887 (PMC4298719; doi:10.1038/srep07887)
Supplement: Supplementary Information [file srep07887-s1.doc]

# Supplementary Information for:

**Highly Sensitive and Multifunctional Tactile Sensor Using Free-standing ZnO/PVDF Thin Film with Graphene Electrodes for Pressure and Temperature Monitoring**

James S. Lee^1*^, Keun-Young Shin^1*^, Oug Jae Cheong^1^, Jae Hyun Kim^2^ and Jyongsik Jang^1,§^

^1^World Class University program of Chemical Convergence for Energy & Environment, School of Chemical and Biological Engineering, Seoul National University, 151-742, Korea

^2^Manufacturing Technology Team, Infra Technology Service Center,

Device Business, Samsung Electronics, San #16 Banwol-Dong, Hwasung-City, Gyeonggi-Do, Korea.

^§^Correspondence and requests for materials should be addressed to J.J. (jsjang@plaza.snu.ac.kr).

[*] E-mail: jsjang@plaza.snu.ac.kr

Tel.: +82-2-880-7069

Fax: +82-2-888-1604


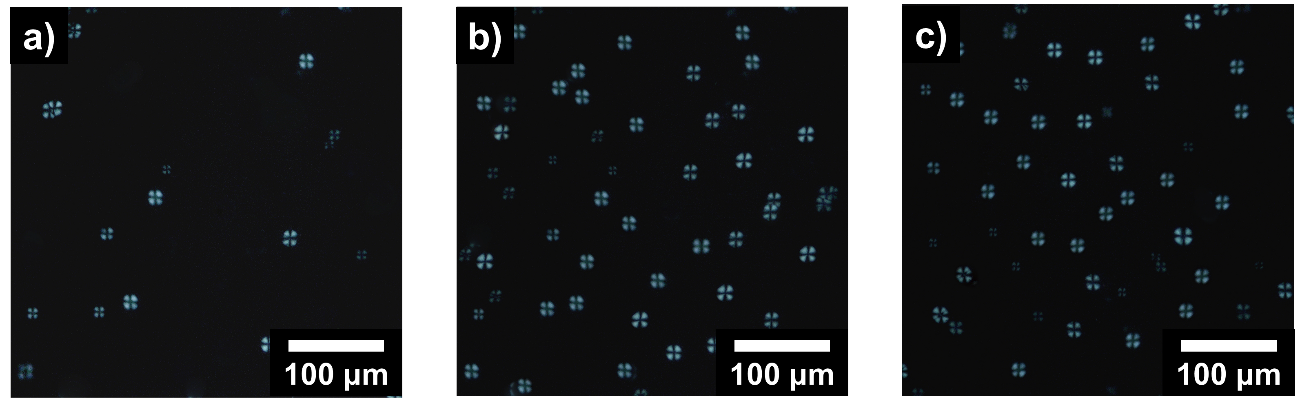


**Figure S1.** Polarized optical microscopy image of a) pristine PVDF, b) PVDF/ZnO rods, c) PVDF/ZnO disks.

The polymer PVDF and semi-conductor ZnO are one of representative dielectric materials which is a substance that inducing electricity by dielectric polarization under electric fields. Especially, the considerable matter of ZnO in our experiment is behavior as role of fillers in the PVDF, whereas matter of conductivity since it is not modified as a transistor. In our previous paper, we reported the function of high permittivity nanofillers for PVDF^1^. As presented figure 4a in the manuscript, it is denoted that ZnO enhanced the crystallinity of PVDF β phase. Also, Fig S1. shows that observed polarized optical microscopy morphology (POM) of the crystal growth of PVDF with various ZnO fillers. All samples were isothermally crystallized at 170 °C and maintained for 240 s, and it cooled down to observe crystal growth morphology. During poling process of film, high external voltage was applied on the film and polarized ZnO induced β phase of PVDF. Therefore, regardless of ZnO morphology, it is clarified that ZnO acts as nuclei for PVDF crystallization. However, the sensitivity of detecting pressure capability showed difference because of aspect ratio of ZnO rods and disks. Since free-standing ZnO rod had large aspect ratio (AR = 3.5) than disk (AR = 0.3), polarization at the each upper and lower terminals are easily occurred by applied vertical pressure^2^. Considering these results, ZnO improves permittivity of PVDF, thus enhanced PVDF with ZnO rod enable to fabricate a highly sensitive tactile sensor.

1. Lee, J. S., Shin, K.-Y., Kim, C., Jang, J. Enhanced frequency response of a highly transparent PVDF–graphene based thin film acoustic actuator. *Chem. Commum*. **49**. 11047-11049 (2013).
2. Wang, Z. L., Song, J. Piezoelectric Nanogenerators Based on Zinc Oxide Nanowire Arrays. *Science*. **312**. 242-246 (2006).

**Table S1.** The dielectric parameters of the PVDF film and the two composite ZnO/PVDF films.

| Sample | *ε'* ^a^ | *Δε* ^a^ | 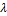/[μs] ^b^ |
| --- | --- | --- | --- |
| Pristine PVDF thin film | 11 | 14 | 159 |
| PVDF/ZnO nanodisk | 121 | 136 | 130 |
| PVDF/ZnO nanorod | 220 | 226 | 121 |

a Data calculated using the Havriliak-Negami and Fourier transform relationship.

b Data were obtained via the interfacial polarization response relaxation time.

**Table S2.** Relevant dielectric parameters at 10^4^ Hz for PVDF/ZnO nanodisk composite with various filler contents.

| Sample | *ε'* ^a^ | *Δε* ^a^ | 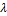/[μs] ^b^ |
| --- | --- | --- | --- |
| PVDF film based on 25 wt% seed solution | 205 | 201 | 128 |
| PVDF film based on 30 wt% seed solution | 220 | 226 | 121 |
| PVDF film based on 35 wt% seed solution | 217 | 217 | 122 |

a Values were calculated by Havriliak-Negami and Fourier transforms relationship

b Values were obtained by interfacial polarization response by relaxation time.


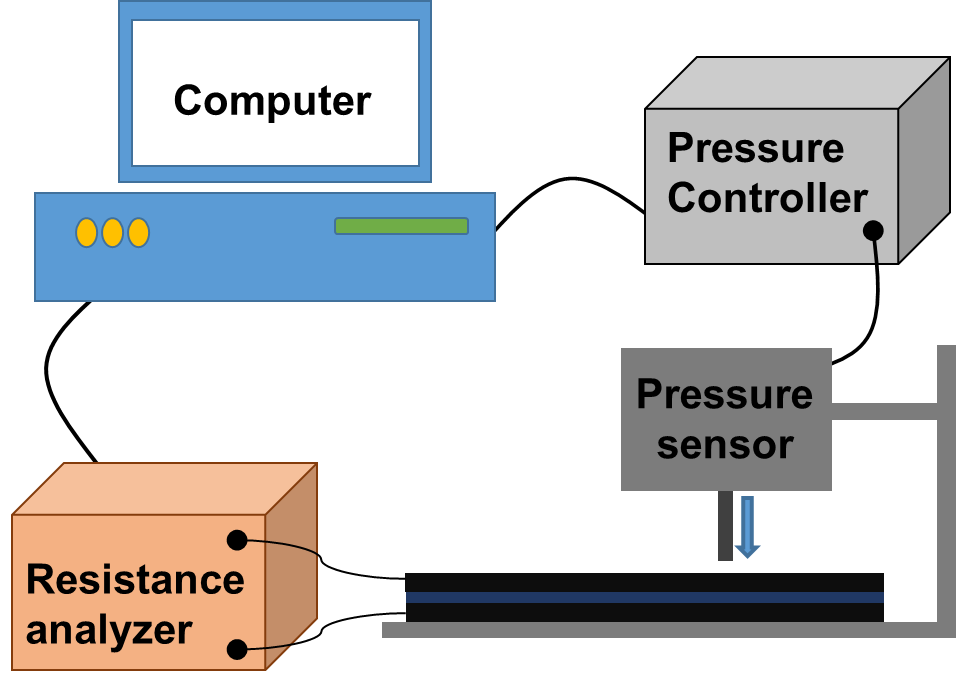


**Figure S2**. Schematic illustration of the pressures sensing measurement which consists of a pressure controller, pressure gauge sensor and computer based resistance analyzing interface.


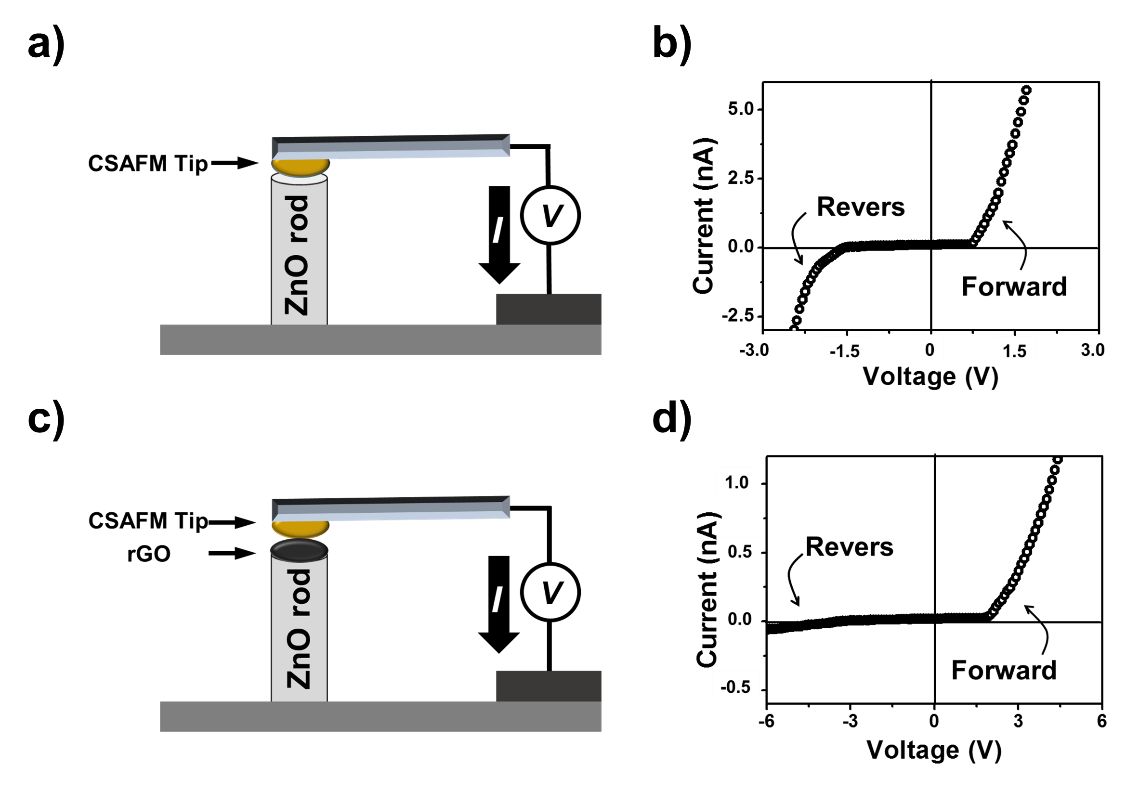


**Figure S3.** Schematic illustration of (a) current-sensing AFM method and (b) I-V characteristic curve of a pristine ZnO rod. (c) and (d) for rGO with ZnO rod.

We evaluated I-V characteristic curves of bare ZnO nanorods and ZnO nanorods with rGO electrode by current-sensing AFM (CSAFM) at 300 K in Fig S3^3,4^. The I-V curves of pristine ZnO nanorods were evaluated by CSAFM (Fig S3a.). As a result, the point contact on pristine ZnO nanorods showed nonlinear and asymmetric behavior as respected under contact force of 30 nN. Moreover, it has a turn-on voltage of 0.75 V to 1.1 V for the forward bias and reverse bias voltage of -2.1 V that results from rectifying contact in Fig S3b. Also, improved electrical characteristics of ZnO with rGO deposition was obtained in the same manner as shown in Fig S3c and a typical I-V curve between CSAFM tip and rGO layer was observed in Fig S3d which the rectifying behavior in I-V characteristics results in Schottky contact.

1. Huang, Y. *et al.* Logic Gates and Computation from Assembled Nanowire Building Blocks. *Sience.* **294**. 1313-1317 (2001).
2. Wang , Z. L., Song, J. Piezoelectric Nanogenerators Based on Zinc Oxide Nanowire Arrays. *Sience.* **312**. 242-246 (2006).


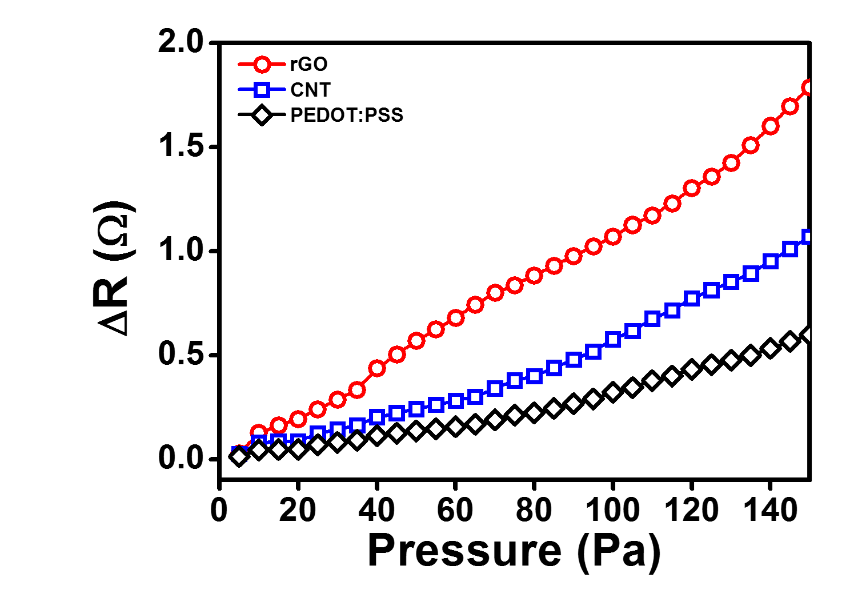
**Figure S4.** Change in resistance of PVDF/ZnO rods composite film with three different electrodes by applied various pressure.


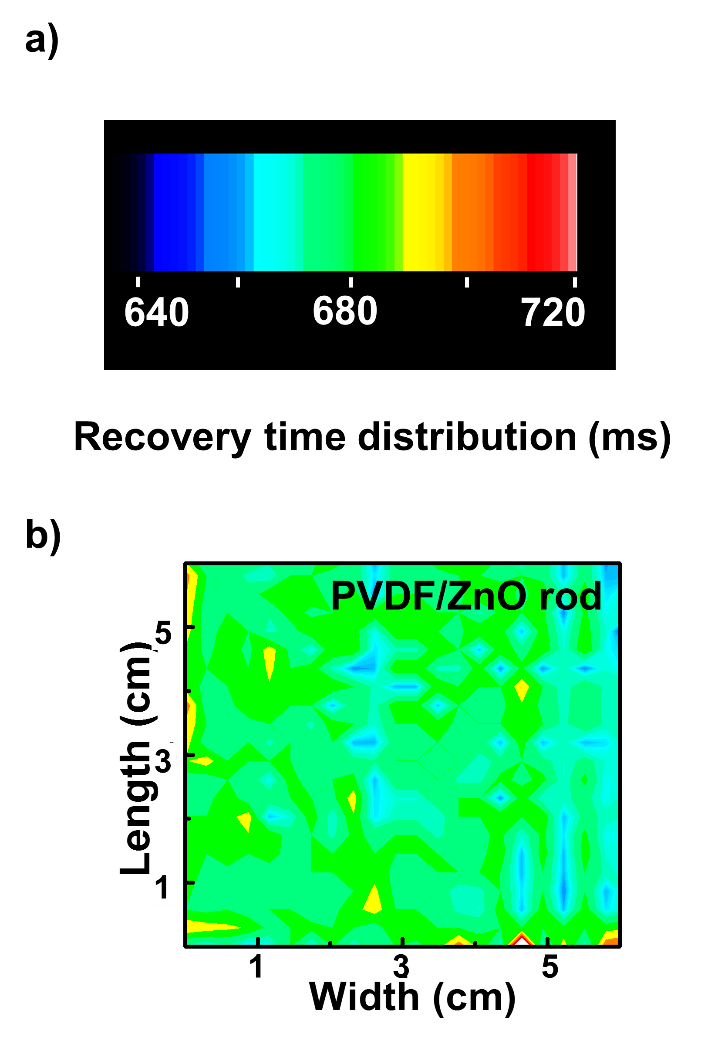


**Figure S5**. (a) The spatial variation of recovery time distribution by mille seconds. (b) . The fabricated PVDF/ZnO rod based film (effective area of 6 × 6 cm^2^) was divided into 144 regions (0.5 × 0.5 cm^2^) and 70°C of Pt weights were used to apply a pressure of 30 Pa at each division, one region at a time, and it shows that 94 % of reliable uniformity by visual color distribution for recovery time of 679~682 ms which represents surface temperature of 70°C.
